# Doctoral level research and training capacity in the social determinants of health at universities and higher education institutions in India, China, Oman and Vietnam: a survey of needs

**DOI:** 10.1186/s12961-017-0225-5

**Published:** 2017-09-02

**Authors:** Farhad Ali, Arun Shet, Weirong Yan, Abdullah Al-Maniri, Salla Atkins, Henry Lucas, for the ARCADE consortium

**Affiliations:** 1 Health Advisor, Save the Children, Delhi, India; 2 Hematology Research Division, St. Johns Research Institute Department of Medical Oncology, St. Johns Medical College and Hospital, Sarjapur Road, Bangalore, 560034 India; 3 Department of Public Health Sciences, Karolinska Institutet, Tomtebodavägen 18A, 17177 Stockholm, Sweden; 4 Road Safety Research Programme The Research Council, Muscat, Oman; 5 Institute of Development Studies, UK Library Road, Brighton, BN1 9RE United Kingdom; 6 Faculty of Medicine and Life Sciences, University of Tampere, PO Box 100, Tampere, Finland

**Keywords:** Social determinants of health, Postgraduate education, Public health, ARCADE, Research capacity building, Asia

## Abstract

**Background:**

Research capacity is scarce in low- and middle-income country (LMIC) settings. Social determinants of health research (SDH) is an area in which research capacity is lacking, particularly in Asian countries. SDH research can support health decision-makers, inform policy and thereby improve the overall health and wellbeing of the population. In order to continue building this capacity, we need to know to what extent training exists and how challenges could be addressed from the perspective of students and staff. This paper aims to describe the challenges involved in training scholars to undertake research on the SDH in four Asian countries – China, India, Oman and Vietnam.

**Methods:**

In-depth interviews were conducted with research scholars, research supervisors and principal investigators (n = 13) at ARCADE partner institutions, which included eight universities and research institutes. In addition, structured questionnaires (n = 70) were used to collect quantitative data relating to the courses available, teaching and supervisory capacity, and related issues for students being trained in research on SDH. Simple descriptive statistics were calculated from the quantitative data and thematic analysis applied to the qualitative data.

**Results:**

We identified a general lack of training courses focusing on SDH. Added to this, PhD students studying related areas reported inadequate supervision, with limited time allocated to meetings and poor interpersonal communication. Supervisors cited interpersonal communication problems and student lack of skills to perform high quality research as challenges to research training. Further challenges reported included a lack of research funding to include SDH-related topics. Finally, it was suggested that there was a need for institutions to define clear and appropriate standards regarding admission and supervision of students to higher education programs awarding doctoral degrees.

**Conclusions:**

There are gaps in training for research on the SDH at the surveyed universities and research institutes, which are likely to also be present in other Asian countries and their higher education institutions. Some of the barriers to high quality research and research training can be addressed by improved training for supervisors, clearly defined standards of supervision, finances for student stipends, and increased use of information and communication technology to increase access to teaching materials. Increased opportunities for online learning could be provided.

## Background

Social determinants of health (SDH) are the conditions in which people are born, grow, work, live and age, and the wider set of forces and systems shaping the conditions of daily life [1, 2]. High quality research on SDH and the mechanisms underlying health inequity is essential to improving health, especially of those who are vulnerable and underserved. It can also contribute towards evidence-based health policy [3] to improve healthcare [4]. However, SDH, by their very definition, are complex and researching their effects requires skills that are not readily available in low- and middle-income country (LMIC) contexts [5, 6]. Researchers have indicated that, in LMIC settings, the quantity and quality of research findings is poor [7] and of limited public health relevance [8]. The focus of research is also not on SDH issues; in China, inequitable health outcomes are common [9], but research tends to focus on access to healthcare. Improving the national capacity to conduct research on SDH in China is increasingly being recognised as a felt need [3]. The need to develop national research capacity is shared by other Asian countries such as Pakistan [10] and Bangladesh [11].

Given the above challenges, research capacity building is needed in Asia. High quality SDH research encompasses investigation into the mechanisms by which social conditionings cause ill-health and structures of primary care where medicines converge with public health [12]. This research capacity building [13] should include developing skills and confidence, supporting linkages and partnerships, ensuring that research is close to practice, develop appropriate dissemination, invest in infrastructure, and build elements of sustainability and continuity. These skills are central to advancing the well-being of populations and improving health [4], including economic benefits [14].

However, capacity building efforts need to be structured and well planned. They need to be structured around the identification of relevant research problems, the dissemination of research findings to a large and diverse audience, especially thought leaders and key policymakers, and have a clear focus on the application of those findings [15]. This requires sustained investment from universities in LMICs [16]. Several initiatives have already been undertaken to enhance the quality of research training in the area of SDH and health equity in Asian countries, including the ‘INDEPTH Training and Research Centres of Excellence’ (INTREC) project [17], which addressed capacity building for SDH. This paper describes the needs assessment for another capacity building project, the ARCADE RSDH (African/Asian Regional Capacity Development – Research in Social Determinants of Health) consortium [18].

The needs assessment conducted was considered the first step of capacity building, namely identifying the resources and training already available at partner institutes on SDH research and potential gaps that needed addressing. This paper reports on a cross-sectional study conducted at partner universities to survey existing SDH-related training, supervision and infrastructure. We describe the results of this survey and identify ongoing challenges at partner institutions.

## Methods

This was a mixed methods study, utilising both qualitative and quantitative methods. The research was designed as a needs assessment within the ARCADE consortium and conducted from February 2012 to September 2012. The needs assessment was performed in the areas of courses available pertaining to SDH, research projects in SDH, research funding for PhD Scholars in the area, access to research publications and online courses, and supervisors’ capacity to support research scholars in research methodology and research management capacity.

### Participants

Key individuals in partner institutes in ARCADE RSDH formed the participants to these studies. The research was conducted in the Asian partner countries of ARCADE RSDH, namely China, India, Vietnam and Oman. The universities or research institutions (henceforth referred to as research institutions) composing this consortium were invited to participate in the study and these institutions have been conducting research on topics relevant to SDH. The participants were administrative officers in administrative offices at the partner institutions, staff from dean’s offices, PhD supervisors, PhD students and principal investigators of the project. Table 1 below details the countries, institutions, institution types and the department in which data collection was conducted.

**Table 1:** Country, institution type and data collection department of partner institutes

Country	Institution	Institution type	Department data collection was conducted
China	Tongji Medical College	University	Community medicine
China	Beijing Normal University	University	Medical college
China	Zhejiang University	University	Medical college
Vietnam	Hanoi Medical University	University	Social and preventive medicine
Oman	Sultan Qaboos University	University	Medical college
India^a^	Indian Institute of Health Management Research	Independent research and training institution	Institution
India^a^	Ruxmaniben Deepchand Gardi Medical College	Independent medical college	Community medicine
India^a^	Saint John’s National Academy of Health Sciences	Catholic Bishop Church of India Society of Medical Education	Medical college

^a^None of the Indian institutions were part of a university

### Data collection and sampling

The team developed an electronic questionnaire informed by an existing questionnaire implemented in ARCADE Health Systems and Services Research [19] to collect data from eight partner institutions involved in the research training in SDH. The survey instrument consisted of closed- and open-ended questions aimed to identify the courses available on SDH, teaching and supervisory capacity on the SDH and associated research methods, and other issues that could contribute towards capacity building in SDH. Table 2 delineates the areas of enquiry. It is based on a framework that supports a systemic approach where focus remains on the entire causal chain and interrelations rather than the discrete nature and function of each component in the SDH research and training [12]. The questionnaires were sent via email to the principal investigators of each institution, who were asked to identify the persons most appropriate to complete them. Reminders were sent to institutes that had not responded in the allotted time. Table 2 details the number of respondents to the questionnaire and the topics that they informed. Responses were received from six Administrative Offices and seven Deans’ offices. The responses to the questionnaires were collated onto Excel when received by the study team.

**Table 2:** Number of respondents to the surveys and interviews with topics addressed

	Number	Topics addressed
Principal Investigator of ARCADE Project	5	Courses available in social determinants of health (SDH)
Administrative Office	6	Department working on SDH issues Faculty positions Students pursuing PhD
Dean’s Office	7	Research grant and projects Institutional review board Supervision training of research supervisors
PhD Students	35	Issues and challenges in completing PhD work Learning facilities Suggestions to improve the learning environment
PhD Supervisors	31	Challenges in completion of research Ways to address the challenges

Key informants were deans and principal investigators, most of whom had had supervision experience but also a broader view of capacity building at their institutions. They were invited to participate by telephone or email. The interviews conducted were semi-structured, using a guide developed by the team. The guide used with supervisors focused on challenges and potential solutions relating to research and teaching capacity on SDH, research funding and, for the students, their motivation to pursue PhD training. We continued collecting data until we considered that data saturation was achieved. In total, 35 PhD students, 31 PhD Supervisors and 5 principal investigators of ARCADE Project were interviewed. All interviews were conducted in English via Skype or in person. A research associate in the project transcribed the interviews.

### Data analysis

Thematic analysis was used to assess the qualitative data, focusing on the manifest level of analysis [20]. The research associate read and re-read the transcripts, identifying codes freely. Following this, transcripts were coded, and codes generated into categories and themes. Table 3 depicts an example of the analysis process. The categories and themes were discussed within the research team and amended by referral back to the data. No qualitative data analysis programme was used.

**Table 3:** Example of analysis

Theme	Category	Code	Quote
Financial concerns	Support during study	Conflict between needing to secure a living and doing a PhD	*I have seen many people not being able to do PhD because they have not got simultaneously funding to stay in the institute and work*
	Lack of research funding impacting on studies	Challenge of insecure research funding	*Ensuring funding for research and you have support for life. There are certain uncertainties and these are real challenges*
Quality of supervision	Time constraints to do research	Supervisor demands interfering with studies	*… my supervisor wants to see me every single day … every time we meet there will be changes* … [for example] *look at the physical activity* [then] *cancel it … when a lot of work will have been done …*
	Quality of supervision	Good supervisor is important for study	*Sometimes one is unlucky and does not get a good supervisors. So getting a good supervisor is very important*

Given the limited sample size and the intention of using the findings from the quantitative data to support the interpretation of the interviews and FGDs, analysis of the questionnaires was limited to calculating simple frequency distributions using Excel. The results below present a combination of both the qualitative and quantitative data.

### Ethics

Ethical clearance for the research was provided by Institutional ethics Committee, St. Johns National Academy of Health Sciences. The ethical approval reference number for this study was ‘IEC Study Ref No: 64/2012’. The study was explained to the participants, and the confidentiality and anonymity of the responses was emphasised. All interview participants provided written informed consent.

## Results

### Available infrastructure and content for training in SDH

The first part of our survey focused on the available support infrastructure for conducting research and on the SDH-specific content that was available in courses.

#### Courses available supporting SDH research

A list of courses relevant to SDH that were available at partner institutes was compiled using online forms. The courses were mainly in the areas of health economics, epidemiology, biostatistics and public health, the last emphasising community health, health management and health promotion. There were few courses on aspects of methodology that may be particularly relevant for SDH research, such as qualitative methods, mixed methods and literature reviews. The only institution from the ARCADE collaboration that offered courses on social sciences and social medicine was Beijing Normal University (BNU) in China, while only the Indian Institute of Health Management Research (IIHMR) in India ran a course on international health. Overall, Chinese institutions had a wider variety of courses relevant to SDH when compared to those in the other countries. Though SDH-related courses were sometimes available for students, many of them noted (21 of 35) that the modules did not sufficiently take into account the geographic and cultural aspects of their region.

**Fig. 1:**
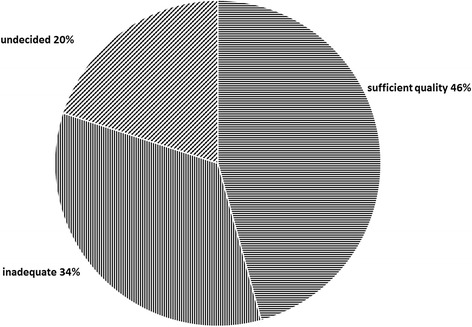
Student assessment of the quality of available learning modules

Almost 50% of doctoral students (16 of 35), felt that the learning modules available at their universities were adequate and of sufficient quality (covering range of socioeconomic, cultural and political issues pertaining to health), while another 12 declared them inadequate and 7 were undecided (Fig. 1).

It seemed that there was a need for training on the practical aspects of conducting research, and in particular on writing up findings for publication. A male student commented:

“*In general there should be more guidance and emphasis on research paper writing, writing abstracts and posters for workshops, accessing various online journals and submission of papers and articles to various journals. On these topics, trainings should be encouraged*.”

Reflecting this student’s comment, training on disseminating research findings to policymakers was available only at BNU, Hanoi Medical University (HMU), IIHMR and Sultan Qaboos University (SQU). At IIHMR, this topic is one of those addressed in the annual course on research methods. However, several other institutions reported that students received instruction in this area despite the lack of a formal training module.

As part of surveying the courses available, students were also asked how they would prefer to receive training. Students had mixed opinions regarding the mode of delivery of the course modules that they took. Some favoured formal classroom-based teaching while others thought that more flexible approaches including computer-based self-study were preferable. There was considerable support (21 out of 35) for the use of free online course modules over the Internet. Making such courses more readily available was seen by interviewees as one way to encourage potential candidates to pursue doctoral studies.

#### Research grant management and institutional review boards (IRB)

Research grant management systems and institutional review boards can contribute toward obtaining research funding grants and maintaining research quality. This was particularly challenging as it required considerable interdepartmental coordination and cooperation. This further became challenging due to competing priorities, timelines and resources available with different department due unavailability of structures that pull these department together. The institutions surveyed had different approaches to managing grants, from supervisor-managed to centralised management systems (Table 4). Notably, only Ruxmaniben Deepchand Gardi Medical College (UCTH) in India reported having a central grants management system. As can be seen from the table, there is great variety across institutions in terms of the number of research grants awarded to students, from 0 to 150 per institution. Some of the institutions, for example IIHMR in Jaipur in India, reported that they did not award grants to students, who were instead funded by external organisations. All of the institutions had institutional review boards that met with varying frequency, from every two weeks to once a year.

**Table 4:** Grant management systems and grants received by students

	ZJU, China	TJMC, China	BNU, China	HMU, Vietnam	IIHMR, India	UCTH, India	SQU, Oman	SJNAHS, India
Grant Management System	Supervisor applies for and manages grants	Researchers relevant to the funds	No fixed system and funding mainly in the areas of poverty, health and social development	No specific system	Students involved in externally funded projects	Grants managed centrally and most funding is external	Contract between researcher and university; mainly internal funds and donations	Not centralised Researcher applies for funds and defines budget
Student Research Grants 2007–2012	No specific PhD grants; students join ongoing projects	24	150	4	0	6	2	3
IRB existence	Yes	Yes	Yes	Yes	Yes	Yes	Yes	Yes
Frequency of IRB meeting	Once a year	Needs based	Needs based	Once a month	Needs based	Not fixed	Every 2 weeks	Once a month

*BNU* Beijing Normal University, *HMU* Hanoi Medical University, *IIHMR* Indian Institute of Health Management Research, *IRB* Institutional review board, *UCTH* Ruxmaniben Deepchand Gardi Medical College, *SJNAHS* Saint John’s National Academy of Health Sciences, *SQU* Sultan Qaboos University, *TJMC* Tongji Medical College, *ZJU* Zhejiang University

#### Current practice of SDH training at partner universities

The second major focus of the survey was on the practice of SDH training or research training at the partner institutes. We focused on the available training for supervisors, and supervisor and student perceptions of practices.

#### Training of supervisors and mentors

Formal training of staff to undertake the supervision and mentoring of doctoral students was undertaken at four of the studied institutions: Tongji Medical College (TJMC) and BNU in China, HMU in Vietnam and SQU in Oman. The length of this training varied widely from a few hours to 1 week and it was offered at least once in each academic year. None of the three Indian institutions (IIHMR in Jaipur, UCTH in Ujjain, and Saint John’s National Academy of Health Sciences (SJNAHS in Bangalore) provided such training. Sporadic training as part of international workshops has been given at some of the institutes (SJNAHS). The supervision and mentoring training lacked management aspects, which were considered essential by participants for effective interdisciplinary research work when working with multiple stakeholders having competing priorities and interests.

#### PhD supervisor perspectives

Perhaps reflecting different levels of training for supervisors, as would be expected in a collective of institutes from 4 different countries, the practices of supervisors varied widely across institutes. Generally, doctoral student supervisors mentored 1 to 5 students, with 9 of 31 supervising 1 to 3 students. However, some supervisors (4 of 31) supervised 9 or more students concurrently. Most supervisors engaged with students on a monthly basis (17 of 31), while only six met with their students weekly. In one case, students and supervisors interacted once every 3 months or less often. The number of meetings appears to be entirely at the discretion of the supervisors. One student found that the demands of her supervisor, whom she held in high regard, for very frequent meetings made it very difficult to make progress:

“*… my supervisor wants to see me every single day … every time we meet there will be changes …* [for example] *look at the physical activity* [then] *cancel it … when a lot of work will have been done …*”

Supervisors reported a number of challenges in supervision, including students displaying limited knowledge of their topic, inadequate research skills, carelessness and a lack of initiative. Interestingly, a student from Oman, who came from a non-medical background, reinforced the concern with relevant skills:

“*…basically I don’t have the background that is needed for the College of Medicine … I wish there were training in skills … when I entered here … based on the interviews they thought that I don’t need any training … in PhD we don’t have courses … there are so many things you are not aware unless you are in the field …*”

This student also pointed out that PhD students had often spent many years in employment since leaving university. While the knowledge of the wider world may be a valuable asset in terms of research on the SDH, it is to be expected that they will need help in meeting the very different challenges encountered in academic research at the highest level. Supervisors also felt that interpersonal communication between supervisors and students could be improved upon. Some of the supervisors indicated that students suffered from a lack of financial and other resources, agreed that supervision was not always of good quality and suggested that students could benefit from organised forums in which they could discuss issues of mutual interest. A male professor said:

“*During the PhD training they may not have adequate resources… funding to perform research…they may not have adequate guidance….they may not have adequate mentorship. They may not have a forum in which they are able to discuss with each other and try resolve some issues or discuss problems*.”

PhD supervisors seemed to think that larger underlying issues, specifically inadequate research funds and unavailability of student stipends, posed serious challenges to some students. These were seen as major demotivating factors, and were linked by supervisors to the failure by many students to focus sufficiently on their doctoral program, given that they were distracted by engagement in other income generating activities. Other factors mentioned in the interviews included the difficulties of accessing research materials (such as full text research articles), the limited ability of many new students to produce coherent and readable written outputs and time-consuming family responsibilities.

#### Doctoral students’ perspectives of research training

**Fig. 2:**
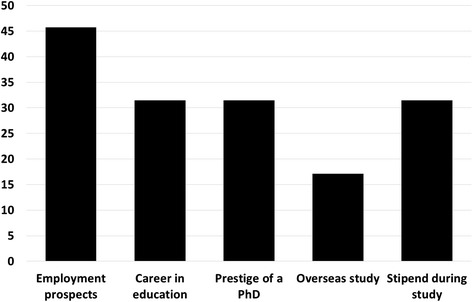
Issues influencing student decisions to apply for a PhD course

The first issue surveyed was the reasons for students to join a PhD programme. The most frequently mentioned motivation was to obtain a highly paid job after graduation (16 of 35). Eleven students mentioned wanting to pursue a career in tertiary education and research, and saw the PhD as a necessary first step. A similar number were inspired by the prestige associated with a PhD degree. The opportunity to study outside the home country was important for six students and the availability of a stipend during study was mentioned by 11 students (Fig. 2).

The students suggested that motivation to apply for doctoral programs would be increased if research grants were available (22 of 35) and scholars were provided with an adequate monthly stipend (20 of 35). A male student from India emphasised the importance of financial factors:

“*I was lucky to get the funding for my project as well as for myself, through the Erasmus program. But not everybody is lucky like that. I have seen many people not being able to do a PhD because they have not got funding.*”

Most students (22 of 35) reported that becoming a doctoral student had not been easy. Though only two students reported multiple applications, many indicated that they had delayed the start of their doctoral training; 11 said that this was due to existing work commitments, and 5 that they could not find a suitable training program. Other challenges included lack of awareness of training opportunities and family commitments.

Most (21 of 35) students considered that the most important factor that would contribute to a successful PhD was the level of commitment by supervisors. Interestingly, while they expressed a general concern with the quality of supervision, only a few students reported problems with their own supervisors. The qualitative data suggested that students considered supervisors’ commitment as the most important issue in terms of pursuing their doctorate.

Reflecting this, a female student from India said:

“*… sometimes one is unlucky and does not get a good supervisor. So getting a good supervisor is very important…*”

Students suggested that more time from supervisors and a more systematic approach to research work would considerably improve the PhD training experience. One student from Oman, for example, reported that:

“*It is very difficult to meet with our supervisors because they are very busy with their teaching and with their clinics.*”

One concern raised by some was that they did not have sufficient voice in the selection of the PhD topic, which tended to be imposed by their supervisors. In many cases, the students would receive multiple invitations to work on a particular topic and it was difficult for them to make a serious assessment as to which was most appropriate. As one reported:

“*… they should give a chance for the student to decide which lecturer she wants to work with, rather than the lecturer approaching him or her …* [saying] *OK, you work with me we will do this or finish this …*”

The salary enhancement offered to staff for taking on PhD students was seen as problematic by this student, they saw it as encouraging a bidding process which could be very confusing. Besides the importance of PhD supervisors, other factors cited by the students as contributing to a successful PhD included an ability to focus on the doctorate, access to training materials and monthly stipends that alleviated distracting financial concerns. Many of the students also reported that engaging in a doctoral programme was challenging. Some (8 of 35) had experienced serious delays in completing their doctorate; 14 students said that they faced pressure to seek employment instead. Other students reported challenges that included inadequate reading material (10 of 35) and inadequate internet facilities (9 of 35) constraining learning and knowledge building of the students on the topics related to SDH. Some other challenges that students faced comprised financial constraints (8 students) and family responsibilities (10 of 35). This last item was seen as a major concern, especially for female students, a view reinforced by one principal investigator, who reported that, in his personal experience:

“*…parents … don’t respect that their children have to study … when the children come home and need to study … the parents …say don’t do that, you have to care for your mother you have to care for your grandmother, you have to help your father, you have to help your brother…*”

Finally, students were asked to suggest how PhD training could be improved. Their suggestions included the need for increased support on how to write research papers, though some argued that too much weight was placed on the ability to produce well-crafted publications and that this should not be a mandatory requirement. Other issues raised were related to providing greater access to course modules available on the internet, increasing the level of coursework in doctoral programs and the allocation of credits for online coursework. One commented:

“*I think if new courses are available online that will benefit and help students to complete their PhD timely and meet the requirements of the research degree. Such courses are very much needed. The content is most important. People will participate only if the content is good*.”

Some students (mainly from China) also requested more English preparation courses to improve their language skills, and exchanges of staff and students across countries were considered potentially helpful.

## Discussion

Numerous constraints that inhibit research on the SDH among the participating ARCADE RSDH institutions are described above. Such constraints probably apply to many similar institutions of research and higher education in Asia and elsewhere [19], limiting the impact of research in SDH. While a number of North–South collaborations have attempted to address this situation (e.g. [6, 16]), previous attempts at research capacity building have adopted a relatively narrow focus, typically implementing training on specific areas or methods to selected trainees linked to particular research projects [21], or establishing joint degrees between institutes [22]. However, from a strategic viewpoint, activities intended to enhancing research capacity in general and particularly in social determinants of health should focus on wider goals, adopt a systems perspective and adapt to specific national or regional contexts [21].

In the different Asian country settings considered here, multiple barriers to the production of high quality research outputs have been identified that need both technical and financial support specifically in SDH research given the interdisciplinary nature of this work [17]. These include a lack of appropriate and context-specific courses that may have long-term effects with regards to lack of confidence and research skills as working professionals, limited funding for student maintenance, limited funding for research activities, varying quality of supervision, grant management practices that fail to incentivise students and challenges of interdepartmental coordination. All of these factors are important to capacity building, as multiple ingredients, not only dependent on students and supervisors, influence the completion of research degrees [23]. Our recommendations for improving research training in SDH in Asian institutions are summarised in Box 1 and discussed in greater detail below.

**Box 1: Key recommendations**

**Table:** 

• Training of supervisors for meaningful engagement with their students • Standards of supervision should be formally defined in line with the country context • Institutions should try to ensure accessible training materials in social determinants of health and public health, possibly in partnership with other national or international organisations • Consider using and crediting blended learning approaches to improve course access • Doctoral research should be professionally managed with clear roles and responsibilities and defined milestones • Use of information and communications technology should be promoted to improve the access to training material • Institutions should try to secure sustainable finances to fund research work and provide stipends to students in order to attract and retain the most capable	

Supervision of PhD students is central to student training [24], and is an an aspect that we found highlighted by both students and their supervisors. As might be expected, students suggested that supervisors allocated insufficient time and failed to address their problems adequately, while supervisors cited student lack of basic academic abilities and disinclination to focus on their work program. Interestingly, a number of students also felt insufficiently prepared for the challenges of the PhD and felt that a more structured training programme at the start of their doctoral studies would allow them to gain the required expertise on specific areas which had not been covered in their initial degree courses. These findings do not differ from general literature on supervision problems, as insecurity among doctoral students is common in many settings, including Sweden [25]. However, an approach involving structured training at the start would seem particularly relevant in terms of SDH research, given the need to attract students from a wider range of disciplines and experiences, particularly if the broader focus discussed above is to be realised.

Both supervisors and students reported that poor communication was a serious barrier to progress. One potential reason for these observations could be a lack of understanding of the essential requirements for effective supervision [26] by both students and supervisors, the latter compounded by the relative absence in partner institutions of formal training on the supervision and mentorship process. In our study, we found that only four out of the eight institutes reported having supervisor or mentor training programs. As the quality of supervision has a direct bearing on the outcome of postgraduate training [26], providing formal training for those supervising doctoral students could greatly benefit partner institutions. This training could focus on the development of the range of positive supervisory characteristics, importantly relationship skills [27]. These skills could include being a guide, teacher, friend, advisor, director and manager who directs study, monitors progress, gives systematic feedback and plans future work [15].

Supervisors also have an important position in enabling the student to become part of the scientific community [24], which is improbable when students are met only every 3 months, as some were in our study. Again, in the case of SDH doctorates, this training should aim to enable supervisors to appreciate the specific needs of students from a range of academic backgrounds and have fewer expectations as to their familiarity with topics such as health systems, epidemiology or statistical analysis.

Given the challenges identified in our study, there is a need for greater attention on a range of procedures for capacity-building, including setting standards for the admission of students to doctoral studies, drafting guidelines for their supervision or even supervising supervisors [28], defining the required minimum frequency of student/supervisor contact and limiting the number of students guided by each supervisor. Acknowledging the input of the supervisor and the impact of students on their workload is also important [29]. In Europe, the European Commission has emphasised the importance of harmonising the standards for academic degrees and for ensuring the quality of courses [30], including arrangements for student supervision.

Inadequate student funding, another major issue raised by this study, was perceived by both students and supervisors as negatively impacting on multiple aspects of doctoral studies in SDH research topics. This issue could reflect the importance placed on SDH topics by country and international funders. Many students postponed the start of their training because of a reluctance to abandon paid employment, and engagement in other income generating activities during training was common. Other authors have also found that faster completion of degrees is related to higher levels of financial support [31]. The low levels of institutional grants given to students suggest that students are dependent on external support, which is difficult to obtain. Supervisors also suggested that students’ effectiveness in studying is compromised by their need to source funding. Given these challenges, in order to build the next generation of competent local scientists, there is a need for advocacy and funding around institution-based grants for doctoral students to motivate both enrolment of highly qualified candidates and ensure their full engagement with their studies. One recent advocacy effort made to the governing body of the Wellcome Trust/DBT India Alliance, a foundation in India, resulted in approval of funding support for research training fellowships for clinicians and public health researchers [32]. Furthermore, having centralised grants management systems and administrators who are familiar with SDH research topics and the specific issues around obtaining research funding in this area, has the potential to contribute to obtaining grants for student support.

Finally, we found that there was a limited availability of courses that address the needs of SDH research at partner institutions. This was not wholly surprising, given previous research [6] and that the importance of this topic has only come to the fore over recent years. However, it does indicate an urgent need to improve the training of young research students in this area, so that they can become the designers and implementers of relevant courses in the future. Joint programs incorporating blended learning courses between northern and southern institutions could be one avenue to address these problems. These programmes could be virtual [33] or large-scale capacity building consortia such as ARCADE RSDH [34]. The potential advantage of the latter is the emphasis on blended learning and mentored seminars.

## Conclusions

The above findings highlight clear gaps in research training capacities at all the eight institutes of research and higher education surveyed in India, China, Oman and Vietnam working together in the ARCADE RSDH project. Overall, capacity for research training at the surveyed institutions was low, particularly in SDH, possibly due to complexities of the subject. The major challenges for PhD training in SDH were supervision capacity and management of interdisciplinary research, as it needs support and mentoring from research guides with different research expertise, funding for students, and courses and materials for SDH education. More attention and effort should be made for improving health research-related capacity, particularly in research on the SDH, at Asian higher education institutions. For this purpose, it is critical that the need to improve the quality of research training is recognised by policymakers and institution leaders.
